# The effects of adding angiotensin receptor neprilysin inhibitors to usual care in patients with heart failure: a protocol for a systematic review of randomised clinical trials with meta-analysis and trial sequential analysis

**DOI:** 10.1186/s13643-019-1173-7

**Published:** 2019-10-31

**Authors:** Emil Eik Nielsen, Joshua Feinberg, Ilan Raymond, Michael Hecht Olsen, Frank Victor Steensgaard-Hansen, Janus Christian Jakobsen

**Affiliations:** 1 Department of Internal Medicine – Cardiology Section, Holbæk Hospital, Holbæk, Region Zealand Denmark; 2 Institute of Regional Health Research, University of Southern Denmark, Odense, Denmark; 3 Centre for Individualized Medicine in Arterial Diseases (CIMA), Odense University Hospital, University of Southern Denmark, Odense, Denmark; 4 Copenhagen Trial Unit, Centre for Clinical Intervention Research, Department 7812, Copenhagen University Hospital, Rigshospitalet, Copenhagen, Denmark

**Keywords:** Heart failure, Angiotensin receptor neprilysin inhibitors, ARNI, Systematic review, Meta-analysis trial sequential analysis

## Abstract

**Background:**

Heart failure is a highly prevalent disease with a global prevalence of 37 million, and the prevalence is increasing. Patients with heart failure are at an increased risk of death and morbidity. Traditionally, patients with heart failure have been treated with a beta-blocker in addition to an inhibitor of the renin-angiotensin-aldosterone system. However, new drugs are currently being added to the recommended guideline therapy. The latest drug to be added combines inhibition of the renin-angiotensin-aldosterone system pathway with inhibiting the neprilysin enzyme and is therefore classified as an ARNI. Our objective is to identify the beneficial and harmful effects of ARNIs in the treatment of patient with heart failure.

**Methods:**

This protocol for a systematic review was undertaken using the recommendations of the Cochrane, the Preferred Report Items of Systematic reviews with Meta-Analysis Protocols, and the eight-step assessment procedure suggested by Jakobsen and colleagues. We plan to include all relevant randomised clinical trials assessing the use of ARNIs in the treatment of patients with heart failure. We will search the Cochrane Central Register of Controlled Trials (CENTRAL), Medical Literature Analysis and Retrieval System Online (MEDLINE), Excerpta Medica database (EMBASE), Latin American and Caribbean Health Sciences Literature (LILACS), Science Citation Index Expanded on Web of Science, Chinese Biomedical Literature Database (CBM), China National Knowledge Infrastructure (CNKI), Chinese Science Journal Database (VIP), and BIOSIS to identify relevant trials. We will also search for grey literature and unpublished trials. Extracted data will be analysed using Review Manager 5, STATA 5, and Trial Sequential Analysis. Our primary outcomes will be all-cause mortality and serious adverse events. We will create a ‘Summary of Findings’ table in which we will present our primary and secondary outcomes, and we will assess the quality of evidence using the GRADE assessment.

**Discussion:**

The present systematic review will have the potential to aid clinicians in decision-making and thereby, benefit patients with heart failure.

**Systematic review registration:**

PROSPERO CRD42019129336

## Background

### Description of the condition

An estimated, 37 million people worldwide have a diagnosis of heart failure [1, 2]. The lifetime risk for developing heart failure is approximately 20% [3]. The prevalence of heart failure is increasing, presumably caused by an increase in life expectancy, improved treatment of acute cardiovascular events, and an increase in the prevalence of the risk factors leading to heart failure [1, 2, 4–7]. Common risk factors for developing heart failure are hypertension, coronary artery disease, diabetes mellitus, and metabolic syndrome [3]. Heart failure represents a considerable health care cost with a cost of more than $30 billion annually or about 2% of the healthcare budget in the USA alone, with an expected increase to about $70 billion in 2030 [8–11].

Heart failure may be viewed as the final common stage of many diseases of the heart with different aetiology [11, 12]. Heart failure may result from disorders of the pericardium (e.g. restrictive cardiomyopathy or chronic pericardial disease) [13, 14], myocardium (e.g. idiopathic dilated cardiomyopathy or myocarditis) [15], endocardium (e.g. infectious endocarditis) [16], cardiac valves (e.g. aortic stenosis or mitral regurgitation) [17], vasculature (e.g. ischaemic heart disease or hypertension) [15], or tachycardia (e.g. atrial fibrillation) [12], or from certain metabolic abnormalities (e.g. endocrine dysfunctions) [11]. Ischaemic heart disease, valvular disease, hypertension, and dilated cardiomyopathy serve as the main causes of heart failure in the majority of patients [18–21]. The left ventricular systolic dysfunction caused by, for example, tachycardia or myocarditis has shown to be reversible either partly or completely [22].

The American College of Cardiology Foundation (ACCF) and the American Heart Association (AHA) define heart failure as “a complex clinical syndrome that results from any structural or functional impairment of ventricular filling or ejection of blood” [19, 23]. For practical purposes, guidelines define heart failure as a clinical syndrome in which signs and symptoms include dyspnoea, fatigue, fluid retention, pulmonary congestion, and peripheral oedema [9, 19]. The heart failure guidelines differentiate between three types of heart failure with systolic dysfunction depending on the level of the left ventricular ejection fraction (LVEF) [20, 23, 24].

- Heart failure with an LVEF of 40% or less is named heart failure with reduced ejection fraction (HFrEF).
- Heart failure with an LVEF of 50% or more is named heart failure with preserved ejection fraction (HFpEF).
- Heart failure with LVEF between 41 and 49% is named heart failure with mid-range ejection fraction (HFmrEF).

It is estimated that HFrEF represents half of the patients with heart failure, while HFpEF and HFmrEF each have a prevalence of 35% and 15%, respectively [11]. However, due to the comorbidities such as hypertension and diabetes, HFpEF might be underdiagnosed [25, 26].

The most commonly used method for categorising the *severity* of heart failure symptoms is either the New York Heart Association (NYHA) functional classification [27] or the ACCF/AHA staging system [23, 28].

### Description of the intervention

#### Treatment of heart failure (usual care)

Guidelines recommend treatment of HFrEF with a beta-blocker in addition to an inhibitor of the renin-angiotensin-aldosterone system (ACE-I or angiotensin II receptor blocker (ARB), with the addition of a mineralocorticoid receptor antagonist in patients who remain symptomatic [3, 24]. In addition, diuretics are used in patients with volume overload, patients with preserved ejection fraction, and patients with decompensated heart failure [3, 24].

#### Angiotensin receptor blocker neprilysin inhibitor

Interventions affecting the natriuretic peptide system have long been of interest to improve treatment in patients with heart failure, due to its effect on promoting natriuresis and vasodilation, which theoretically counteract the negative effects of the increased renin-angiotensin-aldosterone system activation seen in patients with heart failure with reduced ejection fraction [29, 30]. The potential isolated effect of natriuretic peptides has been tested both with the administration of synthetic natriuretic peptides and with drugs inhibiting the enzyme called neprilysin that degrades natriuretic (and other vasoactive) peptides [29]. However, natriuretic peptides have neither shown beneficial effects alone in addition to usual care on clinical outcomes in randomised clinical trials [29, 31] nor in combination with an ACE-I (OVERTURE and IMPRESS studies [32, 33]). The combination of an ACE-I and neprilysin inhibitor later showed in a randomised clinical trial assessing the effects of ACE-I and neprilysin inhibitor in patients with hypertension an increase in angioedema [33, 34]. Therefore, new drugs were developed and approved which combine the inhibition of the renin-angiotensin-aldosterone system pathway with an angiotensin II receptor blocker as well as inhibit the neprilysin enzyme. These new types of drugs are classified as angiotensin receptor blocker neprilysin inhibitor (ARNI) [29].

The European Society of Cardiology recommends ARNIs as a replacement for ACE-I in patients with reduced ejection fraction (EF < 35%) who remain symptomatic (NYHA II–IV) despite optimal medical therapy with ACE-I, beta-blocker, and mineralocorticoid receptor antagonist [24]. The American College of Cardiology/American Heart Association Task Force on Clinical Practice Guidelines and the Heart Failure Society of America makes similar recommendations [23]. The recommendations are primarily based on the PARADIGM trial [35], which randomised 8442 participants with HFrEF (LVEF < 35%) who remain symptomatic despite optimal therapy to sacubitril/valsartan vs. enalapril. The trial was stopped early after a median follow-up of 27 months due to the boundary for overwhelming benefit was crossed.

## Why is it important to do this review

ARNIs are currently recommended in patients with hypertension and in patients with HFrEF, who remain symptomatic. One former meta-analysis assessed the effects of combined neprilysin and renin-angiotensin system inhibition in patients with HFrEF [36]. The combined meta-analysis includes two trials assessing neprilysin in combination with an ACE-I vs. standard therapy and one trial assessing neprilysin in combination with an ARB (ARNI). The meta-analysis including three trials, of which only one assessed the effects of an ARNI, showed a reduced risk of all-cause mortality (OR 0.86, 95% CI 0.79–0.94, *P* = 0.001) [36]. The trial assessing the effects of ARNI compared to enalapril found a reduced risk of all-cause death (HR 0.84, 95% CI 0.76–0.93) [35].

A review assessed the effects of sacubitril in adults with HFrEF [37]. This review included two trials. However, they did not perform any pooled meta-analysis due to the difference in types of heart failure, and all results were based on single trials. The review concluded that sacubitril in combination with valsartan compared with enalapril reduced the risk of cardiovascular death and hospitalisations and improved quality of life [37]. No former systematic review has searched all relevant databases, considered both risk of systematic and random errors, and is up-to-date. Therefore, there is a need for an up-to-date systematic review according to the newest methodology, taking into account both risks of random errors using the Trial Sequential Analysis tools and systematic errors using the Cochrane risk of bias tools [38–40].

The question sought to be answered is: What are the beneficial and harmful effects of ARNIs in patients with heart failure?

## Methods

This protocol for a systematic review has been developed based on the Preferred Reporting Items for Systematic Reviews and Meta-Analysis Protocols (PRISMA-P) guidelines for reporting in systematic reviews and meta-analyses [39, 41] and the Cochrane Handbook [40].

### Criteria for considering studies for this review

#### Types of studies

We will include randomised clinical trials irrespective of trial design, setting, publication status, publication year, and language for assessment of benefits and harms. We will not include cluster randomised trials, quasi-randomised studies, or observational studies. Any non-English papers published in a language not mastered by the author group will be translated by health professional translators.

#### Types of participants

We will include participants with heart failure (as defined by trialists). We will include participants irrespective of age, sex, and comorbidities.

#### Types of interventions

Our primary comparison will be ARNIs in addition to usual care (e.g. beta-blockers and mineralocorticoid receptor antagonists) compared with placebo (or no intervention) and a similar usual care (e.g. beta-blockers and mineralocorticoid receptor antagonists).

Our secondary comparison will be ARNIs in addition to usual care (e.g. beta-blockers and mineralocorticoid receptor antagonist) compared with placebo (or no intervention) and a different usual care compared to the experimental usual care (e.g. ACE-I, beta-blockers, and mineralocorticoid receptor antagonists).

We will accept any co-intervention, if the co-intervention is planned to be delivered similarly to the intervention and control groups.

#### Types of outcomes

For all outcomes, we will use the trial results reported at maximum follow-up.

#### Primary outcomes

- All-cause mortality.
- Serious adverse events. We will define a serious adverse event as any untoward medical occurrence that resulted in death, was life-threatening, required hospitalisation or prolongation of existing hospitalisation, and resulted in persistent or significant disability or jeopardised the patient [42] (dichotomous outcome).

#### Secondary outcomes

- Myocardial infarction (dichotomous outcome)
- Quality of life measured on any valid scale (continuous outcome)
- Non-serious adverse events (dichotomous outcome) (please see above)
- Hospitalisation during follow-up (dichotomous outcome)

#### Exploratory outcomes

- Cardiovascular mortality
- Ejection fraction (continuous outcome)
- Six minutes of walking distance (continuous outcome)
- NT-proBNP (continuous outcome)

### Search methods

#### Electronic searches

We will search the Cochrane Central Register of Controlled Trials (CENTRAL), Medical Literature Analysis and Retrieval System Online (MEDLINE), Excerpta Medica database (EMBASE), Latin American and Caribbean Health Sciences Literature (LILACS), Science Citation Index Expanded on Web of Science, Chinese Biomedical Literature Database (CBM), China National Knowledge Infrastructure (CNKI), Chinese Science Journal Database (VIP), and BIOSIS in order to identify relevant trials. We will search all databases from their inception to the present. We will begin the searches in October 2019. Preliminary search can be found in the Appendix section.

#### Searching other resources

The reference lists of relevant publications will be checked for any unidentified randomised trials. We will contact the authors of included trials, and major pharmaceutical companies involved in the production or sales of angiotensin receptor neprilysin inhibitors, by email asking for unpublished randomised trials. Further, we will search for ongoing trials on the following:

- ClinicalTrials.gov (www.clinicaltrials.gov)
- Google Scholar (https://scholar.google.dk/)
- The Turning Research into Practice (TRIP) Database (https://www.tripdatabase.com)
- European Medicines Agency (EMA) (https://www.ema.europa.eu/ema/)
- US Food and Drug Administration (FDA) (www.fda.gov)
- China Food and Drug Administration (CFDA) (http://eng.sfda.gov.cn/WS03/CL0755/)
- Medicines and Healthcare products Regulatory Agency (https://www.gov.uk/government/organisations/medicines-and-healthcare-products-regulatory-agency)
- The World Health Organization (WHO) International Clinical Trials Registry Platform (ICTRP) search portal (http://apps.who.int/trialsearch)

Additionally, we will hand search conference abstracts from cardiology conferences for relevant trials. We will also consider unpublished and grey literature trials relevant to the review, if we identify such trials.

### Data collection and analysis

We will perform the review following the recommendations of the Cochrane Collaboration [40]. The analyses will be performed using Review Manager [43] and Trial Sequential Analysis [44]. In case of Review Manager statistical software not being sufficient, we will use STATA 16 [45].

#### Selection of studies

Two review authors (EEN and JF) will independently screen the titles and abstracts. We will retrieve all relevant full-text study reports and publications. Two review authors (EEN and JF) will independently screen the full text and identify and record the reasons for exclusion of the ineligible studies. We will resolve any disagreement through a discussion, or if required, we will consult a third author (JCJ). Trial selection will be displayed in an adapted flow diagram as per the Preferred Reporting Items for Systematic Reviews and Meta-Analyses (PRISMA) statement [38].

#### Data extraction and management

Two authors (EEN and JF) will extract the data independently from the included trials. Disagreements will be resolved by a discussion with a third author (JCJ). We will assess the duplicate publications and companion papers of a trial together to evaluate all available data simultaneously (maximise data extraction, correct bias assessment). We will contact the trial authors by email to specify any additional data, which may not have been reported sufficiently or at all in the publication.

#### Trial characteristics

The trial characteristics are bias risk components (as defined below), trial design (parallel, factorial, or crossover), number of intervention arms, length of follow-up, estimation of sample size, and inclusion and exclusion criteria.

#### Participant characteristics and diagnosis

The participant characteristics and diagnosis are number of randomised participants, number of analysed participants, number of participants lost to follow-up/withdrawals/crossover, compliance with medication, age range (mean or median) and sex ratio, rhythm, baseline numbers of cardiovascular risk factors (i.e. diabetes mellitus, hypertension, hyperlipidaemia, or smoking), baseline NYHA class, baseline number of participants with heart failure (subdivided according to ejection fraction), baseline number of participants with valvular heart disease, baseline number of participants with previous myocardial infarction, baseline number of participants with previous revascularisation, and baseline number of participants with previous angina. We will additionally report the proportion of participants in the compared groups who receive beta-blockers, calcium channel blockers, long- or short-acting nitrates, diuretics, angiotensin-converting enzyme inhibitors, angiotensin II receptor antagonists, and/or mineralocorticoid receptor antagonists.

#### ARNI strategy characteristics

The ARNI strategy characteristics are dose of intervention, mode of administration, and duration of administration.

#### Co-intervention characteristics

The co-intervention characteristics are type of co-intervention, dose of co-intervention, duration of co-intervention, and mode of administration.

#### Notes

Funding of the trial and notable conflicts of interest of trial authors will be extracted, if available. We will note in the ‘Characteristics of included studies’ table if the outcome data were not reported in a usable way. Two review authors (EEN and JF) will independently transfer the data into the Review Manager file. Disagreements will be resolved through a discussion, or if required, we will consult with a third author (JCJ).

#### Assessment of risk of bias in included studies

We will assess the risk of bias based on the Cochrane Handbook for Systematic Reviews of Interventions as well as meta-epidemiological studies in our evaluation of the methodology and hence the risk of bias of the included trials [46–52]. We will evaluate the methodology in respect of the following:

- Random sequence generation
- Allocation concealment
- Blinding of participants and treatment providers
- Blinding of outcome assessment
- Incomplete outcome data
- Selective outcome reporting
- For profit bias
- Other risks of bias
- Overall risk of bias

##### Random sequence generation

- Low risk: if sequence generation was achieved using a computer random number generator or a random number table. Drawing lots, tossing a coin, shuffling cards, and throwing dice were also considered adequate if performed by an independent adjudicator.
- Unclear risk: if the method of randomisation was not specified, but the trial was still presented as being randomised.
- High risk: if the allocation sequence is not randomised or only quasi-randomised. These trials will be excluded.

##### Allocation concealment

- Low risk: if the allocation of patients was performed by a central independent unit, on-site locked computer, identical-looking numbered sealed envelopes, drug bottles, or containers prepared by an independent pharmacist or investigator
- Uncertain risk: if the trial was classified as randomised but the allocation concealment process was not described
- High risk: if the allocation sequence was familiar to the investigators who assigned the participants

##### Blinding of participants and treatment providers

- Low risk: if the participants and the treatment providers were blinded to the intervention allocation and this was described
- Uncertain risk: if the procedure of blinding was insufficiently described
- High risk: if the blinding of the participants and the treatment providers was not performed

##### Blinding of outcome assessment

- Low risk of bias: if it was mentioned that outcome assessors were blinded, and this was described
- Uncertain risk of bias: if it was not mentioned if the outcome assessors in the trial were blinded or the extent of blinding was insufficiently described
- High risk of bias: if no blinding or incomplete blinding of outcome assessors was performed

##### Incomplete outcome data

- Low risk of bias: if missing data were unlikely to make treatment effects depart from plausible values. This could be either (1) there were no drop-outs or withdrawals for all outcomes or (2) the numbers and reasons for the withdrawals and drop-outs for all outcomes were clearly stated and could be described as being similar to both groups. Generally, the trial is judged as at a low risk of bias due to incomplete outcome data if drop-outs are less than 5%. However, the 5% cut-off is not definitive.
- Uncertain risk of bias: if there was insufficient information to assess whether missing data were likely to induce bias on the results.
- High risk of bias: if the results were likely to be biassed due to missing data either because the pattern of drop-outs could be described as being different in the two intervention groups or the trial used improper methods in dealing with the missing data (e.g. last observation carried forward).

##### Selective outcome reporting

- Low risk of bias: if a protocol was published before or at the time the trial was begun, and the outcomes specified in the protocol were reported on. If there is no protocol or the protocol was published after the trial has begun, reporting of all-cause mortality and all serious adverse events will grant the trial a grade of low risk of bias.
- Uncertain risk of bias: if no protocol was published and the outcome all-cause mortality and serious adverse events were not reported on.
- High risk of bias: if the outcomes in the protocol were not reported on.

##### For profit bias

- Low risk of bias: if the trial is not financed by a company that might have an interest in a given result
- Uncertain risk of bias: if there is no description of how the trial is financed
- High risk of bias: if the trial is financed by a company that might have an interest in a given result

##### Other risks of bias

- Low risk of bias: if the trial appears to be free of other components (for example, academic bias) that could put it at risk of bias
- Unclear risk of bias: if the trial may or may not be free of other components that could put it at risk of bias
- High risk of bias: if there are other factors in the trial that could put it at risk of bias (for example, academic bias)

##### Overall risk of bias

- Low risk of bias: the trial will be classified as overall ‘low risk of bias’ only if all of the bias domains described in the above paragraphs are classified as ‘low risk of bias’.
- High risk of bias: the trial will be classified as ‘high risk of bias’ if any of the bias risk domains described in the above are classified as ‘unclear’ or ‘high risk of bias’.

These components enable classification of randomised trials with low risk of bias and high risk of bias. The latter trials tend to overestimate positive intervention effects and underestimate negative effects [46–52]. We will classify a trial as being at overall ‘low risk of bias’ only if all bias domains are classified as ‘low risk of bias’. We will classify a trial as being at overall ‘high risk of bias’ if any of the bias domains are classified as ‘unclear’ or ‘high risk of bias’. We will also assess for profit bias.

We will assess the domains ‘Blinding of outcome assessment’, ‘Incomplete outcome data’, and ‘Selective outcome reporting’ for each outcome result. Thus, we can assess the bias risk for each outcome assessed in addition to each trial.

### Measures of treatment effect

#### Dichotomous outcomes

We will calculate the risk ratios (RRs) with 95% confidence interval (CI) for dichotomous outcomes, as well as the Trial Sequential Analysis-adjusted CIs (see below).

#### Continuous outcomes

We will calculate the mean differences (MDs) with 95% CI for continuous outcomes, as well as the Trial Sequential Analysis-adjusted CIs (see below).

### Dealing with missing data

We will, as the first option, contact all trial authors to obtain any relevant missing data (i.e. for data extraction and for assessment of risk of bias, as specified above). We will use intention-to-treat data if provided by the trialists.

#### Dichotomous outcomes

We will not impute missing values for any outcomes in our primary analysis. In two of our sensitivity analyses (see paragraph below), we will impute data.

#### Continuous outcomes

We will primarily analyse scores assessed at single time points. If only changes from baseline scores are reported, we will analyse the results together with the follow-up scores. If standard deviations (SDs) are not reported, we will calculate the SDs using trial data, if possible. We will not use intention-to-treat data if the original report did not contain such data. We will not impute missing values for any outcomes in our primary analysis. In our sensitivity analysis (see paragraph below) for continuous outcomes, we will impute data.

### Assessment of heterogeneity

We will primarily investigate forest plots to visually assess for signs of heterogeneity. We will secondly assess the presence of statistical heterogeneity by chi^2^ test (threshold *P* < 0.10) and measure the quantities of heterogeneity by the *I*^2^ statistics [53, 54].

We will investigate possible heterogeneity through subgroup analyses. Ultimately, we may decide that a meta-analysis should be avoided [40].

### Assessment of reporting biases

We will use a funnel plot to assess reporting bias if ten or more trials are included. We will visually inspect funnel plots to assess the risk of bias. We are aware of the limitations of a funnel plot (i.e. a funnel plot assesses bias due to small sample size). From this information, we assess possible reporting bias. For dichotomous outcomes, we will test asymmetry with the Harbord test [55] if *τ*^2^ is less than 0.1 and with the Rücker test if *τ*^2^ is greater than 0.1. For continuous outcomes, we will use the regression asymmetry test [56] and the adjusted rank correlation test [57].

### Unit of analysis issues

We will only include randomised clinical trials. For trials using crossover design, only data from the first period will be included [40, 58]. Therefore, there will be no any unit of analysis issues. We will not include cluster randomised trials.

### Data synthesis

#### Meta-analysis

We will undertake this meta-analysis according to the recommendations stated in the Cochrane Handbook for Systematic Reviews of Interventions [40] and Keus et al. [59]. Thresholds for statistical significance when assessing the meta-analysis results are insufficiently demonstrated by traditional 95% confidence intervals. Therefore, we will use the eight-step assessment suggested by Jakobsen et al. [60] in order to improve the validation of the meta-analytic results. The eight steps used to validate the results are all validated tools that include (1) meta-analyses results, (2) heterogeneity, (3) multiplicity, (4) calculate required information size (using Trial Sequential Analysis), (5) Bayes factor, (6) using subgroup analyses and sensitivity analyses, (7) publication bias, and (8) assess the clinical significance of the statistically significant review results. We will use the statistical software Review Manager 5.3 provided by Cochrane to analyse data [43]. We will assess our intervention effects with both random-effects meta-analyses [61] and fixed-effect meta-analyses [62]. We will use the more conservative point estimate of the two [60]. The more conservative point estimate is the estimate closest to zero effect. If the two estimates are similar, we will use the estimate with the highest *P* value. We will assess two primary outcomes, and therefore, we will consider a *P* value of 0.033 as the threshold for statistical significance [60]. We will investigate possible heterogeneity through subgroup analyses. Ultimately, we may decide that a meta-analysis should be avoided because of unexpected high heterogeneity [40]. We will use the eight-step procedure to assess if the thresholds for significance are crossed [60]. Where multiple trial groups are reported in a single trial, we will include only the relevant groups. If two comparisons are combined in the same meta-analysis, we will halve the control group to avoid double-counting [40]. Trials with a factorial design will be included.

#### Trial sequential analysis

Traditional meta-analysis runs the risk of random errors due to sparse data and repetitive testing of accumulating data when updating reviews. We wish to control the risks of type I errors and type II errors. We will therefore use Trial Sequential Analysis as a tool for quantifying the statistical reliability of data in the cumulative meta-analysis adjusting significance levels for sparse data and repetitive testing on accumulating data. We will perform Trial Sequential Analysis on the outcomes, in order to calculate the required information size (that is, the number of participants needed in a meta-analysis to detect or reject a certain intervention effect) and the cumulative *Z*-curve’s breach of relevant trial sequential monitoring boundaries http://www.ctu.dk/tsa/ [44, 63–71]. For dichotomous outcomes, we will estimate the required information size based on the observed proportion of patients with an outcome in the control group (the cumulative proportion of patients with an event in the control groups relative to all patients in the control groups), a relative risk reduction of 15% as this is our estimation of a minimally important difference. We use two primary outcomes; therefore, we have adjusted our alpha value to 3.3% accordingly using the adjustment approach suggested by Jakobsen et al. [60]. As secondary outcomes are hypothesis generating, we will use an alpha value of 5%. Most trials use a beta of either 10 or 20%. We will use a beta of 10% in order to minimise the risk of type II error. We will use diversity as suggested by the trials in the meta-analysis. For continuous outcomes, we will in the Trial Sequential Analysis use the observed SD, a mean difference of the observed SD/2, an alpha of 3.3% and 5% for the primary and secondary outcomes, respectively, and a beta of 10%.

### Subgroup analysis and investigation of heterogeneity

#### Subgroup analysis

We will perform the following subgroup analysis when analysing the primary and secondary outcomes:

- Trials at high risk of bias compared to trials at low risk of bias
- Participants with HFrEF, HFmrEF, and HFpEF
- Participants with acute decompensated heart failure compared to chronic heart failure
- Participants with NYHA 1 and 2, compared to NYHA 3 and 4
- Trials sponsored by the industry compared to trials not sponsored by the industry

We will use the formal test for subgroup interactions in Review Manager [72].

#### Sensitivity analysis

To assess the potential impact of the missing data for dichotomous outcomes, we will perform the two following sensitivity analyses on both the primary and secondary outcomes.

- ‘Best-worst-case’ scenario: we will assume that all participants lost to follow-up in the ARNI group have survived, had no serious adverse events, had no myocardial infarction, had a higher quality of life (see paragraph below), and had no adverse events. We will assume the opposite for all participants lost to follow-up in the control group.
- ‘Worst-base-case’ scenario: we will assume that all participants lost to follow-up in the ARNI group have not survived, had serious adverse events, had a myocardial infarction, had a lower quality of life (see paragraph below), and had adverse events. We will assume the opposite for all participants lost to follow-up in the control group.

We will present the results of both scenarios in our review. When analysing the quality of life, a ‘beneficial outcome’ will be the group mean plus two standard deviations (SDs) of the group mean, and a ‘harmful outcome’ will be the group mean minus two SDs of the group mean [60].

We will present the results of this scenario in our review. Other post hoc sensitivity analyses might be warranted if unexpected clinical or statistical heterogeneity is identified during the analysis of the review results [60].

If possible, we plan to conduct a meta-regression based on industry sponsorship.

### ‘Summary of findings’ table

We will create a ‘Summary of findings’ table including our primary and secondary outcomes. We will use the five GRADE considerations (bias risk of the trials, consistency of effect, imprecision (will be assessed using Trial Sequential Analysis) [60], indirectness, and publication bias) to assess the quality of a body of evidence as it relates to the studies which contribute data to the meta-analyses for the prespecified outcomes [60, 73–75]. We will use methods and recommendations described in Chapter 8 (Section 8.5) and Chapter 12 of the Cochrane Handbook for Systematic Reviews of Interventions using GRADEpro software [40]. We will justify all decisions to downgrade the quality of trials using footnotes, and we will make comments to aid the reader’s understanding of the review where necessary. First, we will present our results in the ‘Summary of findings’ table.

## Discussion

This systematic review protocol has several strengths. We have based the protocol on the Preferred Reported Items for Systematic reviews and Meta-Analyses Protocol (PRISMA-P) checklist [39, 41]. We have pre-defined our methodology based on the Cochrane Handbook for Systematic Reviews of Interventions [40], Keus et al. [59], the eight-step assessment as suggested by Jakobsen et al. [60], Trial Sequential Analysis [44], and GRADE assessment [74, 76]. Through our pre-defined methodology, we systematically consider both risks of random errors using the Trial Sequential Analysis and systematic errors using the Cochrane risk of bias tool.

The systematic review will also have limitations. We will pool the data from all trials regarding the treatment of heart failure using ARNIs, thereby potentially giving rise to clinical heterogeneity. Moreover, we have pre-defined several comparisons, subgroup analyses, and sensitivity analysis which increase the risk of type I errors. We may even conduct further subgroup analyses and sensitivity analyses to explain the unexplained heterogeneity. By not searching for all non-randomised studies, we likely overlook harms [77]. If the present review finds solid evidence for benefits, then a more thorough investigation of potential harms seems warranted.

With this systematic review, we seek to provide the clinicians and decision-makers on clinical practice with a reliable evidence regarding the treatment of heart failure using ARNI.

## Data Availability

All data, as well as analysis and plots, will be available in the supplementary material.

## Appendix

### Preliminary search

Search strategy for MEDLINE and EMBASE

1. (ARNI or angiotensin receptor neprilysin inhibitor or angiotensin receptor neprilysin blocker).af.
2. (entrest* or LCZ696 or LCZ-696).af
3. (sacubitril* or AHU377 or neprilysin).af.
4. 1 or 2 or 3
5. (random* or placebo* or randomised clinical trial* or trial* or meta-analys* or meta analys* or blind*).af.
6. 6. 4 and 5

Search strategy for CNKI

(SU=(‘沙卡布曲’+‘沙库巴曲’+‘沙库比曲’+‘沙库必曲’+‘塞克比曲’+‘萨库比尔’)*‘缬沙坦’ OR SU=‘ARNI’+‘entresto’+‘LCZ-696’+‘LCZ696’+‘AHU377’+(‘sacubitril’*‘valsartan’)+‘诺欣妥’) and SU=‘心衰’+‘心力衰竭’+‘心脏衰竭’+‘心功能不全’+‘心脏功能不全’+‘心脏失代偿’+‘心脏代偿失调’ and SU=‘RCT’+‘随机’+‘对照’+‘安慰剂’+‘盲’+‘盲法’

Search strategy for VIP

R=((沙卡布曲 OR 沙库巴曲 OR 沙库比曲 OR 沙库必曲 OR 塞克比曲 OR 萨库比尔) AND 缬沙坦) OR (ARNI OR entresto OR LCZ-696 OR LCZ696 OR AHU377 OR (sacubitril AND valsartan) OR 诺欣妥) AND R=(心衰 OR 心力衰竭 OR 心脏衰竭 OR 心功能不全 OR 心脏功能不全 OR 心脏失代偿 OR 心脏代偿失调) AND R=(RCT OR 随机 OR 对照 OR 安慰剂 OR 盲 OR 盲法)

## Abbreviations

ACE-I: Angiontensin-converting enzyme inhibitor
ARB: Angiotensin II receptor blocker
ARNI: Angiotensin receptor-neprilysin inhibitor
CI: Confidence interval
GRADE: Grading of Recommendations Assessment, Development and Evaluation
HFmrEF: Heart failure with mid-range ejection fraction
HFpEF: Heart failure with preserved ejection fraction
HFrEF: Heart failure with reduced ejection fraction
LVEF: Left ventricular ejection fraction
NT-proBNP: N-terminal pro-brain natriuretic peptide
NYHA: New York Heart Association Classification
RR: Relative risk
SD: Standard deviation
